# Assessment of Oral Health Status and Treatment Needs of School Teachers in Indore City, Madhya Pradesh, India: A Cross-Sectional Study

**DOI:** 10.7759/cureus.42170

**Published:** 2023-07-19

**Authors:** Vrinda Saxena, Pragya Pradhan, Ankita Bhargava, Muhammed Musthafa K.B., Neha Shende, Manoj Jain

**Affiliations:** 1 Public Health Dentistry, Government College of Dentistry, Indore, IND

**Keywords:** oral hygiene habits, oral health survey, periodontal disease (pd), dental caries, school teachers, oral health

## Abstract

Introduction: Teachers are central to the well-being of a community. Being an influential part of society, their role can be broadened to promote oral health and healthy oral hygiene habits. This study was aimed at the assessment of the oral health status and treatment needs of school teachers in Indore City.

Method: A descriptive cross-sectional study was carried out over a period of five months on 470 school teachers working in various government schools of Indore city selected through random sampling technique. The modified WHO Oral Health Assessment Form for Adults 2013 was used to record oral health status and treatment needs, while the WHO Questionnaire for Adults 2013 was used to document oral hygiene practices, dietary habits, and deleterious habits. Data was analyzed using IBM SPSS Version 25 (IBM Corp., Armonk, NY). Chi-square test, independent t-test, and one-way ANOVA were used.

Results: The gender distribution of the representative sample showed female predominance. The prevalence of smokeless tobacco consumption was 5.1% among the study subjects. The mean number of Decayed, Missing, and Filled Teeth (DMFT) was 3.45 ± 3.10, and the mean number of 1.52 ± 2.40 teeth showed the presence of bleeding. Around 37.2% had shallow pockets of 4-5 mm. A mean number of 4.26 ± 1.97 sextants showed 0-3 mm attachment loss. There was a significant association between the frequency and technique of toothbrushing with a decayed number of teeth (p<0.001).

Conclusion: High proportion of dental caries and periodontal disease was seen which could be related to their oral health care-seeking behavior and the impairment related to age changes.

## Introduction

Education is the greatest wealth earned by anyone and Teachers’ are considered the fountain of knowledge. India has a deep-rooted history in the field of Education and has a long tradition of giving special status to teachers. As teachers are the primary source of education, health education, healthful living, a healthy environment, and oral health as well, they are the flag bearer for health-promoting activities [1]. They understand that health goes beyond the popular notion of physical well-being and a universal approach to health is paramount. Teachers, therefore, serve as an important vehicle for health-related interventions.

According to United Nations International Children's Emergency Fund (UNICEF), more than 1.5 million schools, 8.5 million teachers, and 250 million students from diverse socioeconomic backgrounds make up the Indian Education System [2]. This large education network can prove to be instrumental in bridging the gap between oral health education and prevention strategies in India. This is also evident from the concept of Health Promoting Schools as given by WHO in 1995, which is conceptualized to encourage the general health of students, staff, parents, and the community [3]. It could be a cost-effective setup to influence and instill good oral health habits among a wider population group of school children [4].

There is ample literature available measuring the Oral Health Related Knowledge, Attitude and Practice of School Teachers [5-7]. But there is scarce documented evidence when it comes to assessing the Oral Health Status of School Teachers, which is paramount to understanding their oral hygiene habits, dietary habits, and oral healthcare-seeking behavior, further giving an insight into their own oral health. Therefore, this study is undertaken to assess the oral health status and treatment needs of government school teachers of Indore City, Madhya Pradesh, India.

## Materials and methods

A descriptive cross-sectional study was conducted to assess the oral health status and treatment needs, oral hygiene, dietary and deleterious habits among the government school teachers of Indore city. The study ran from May 2022 to September 2022, lasting for a total of five months. Schools included were from both urban as well peri-urban areas of Indore City.

Ethical approval was obtained from the Institutional Ethics Committee at the Government College of Dentistry, Indore. Permissions were attained from the District Education Officer (DEO) for Indore District. Informed consent was obtained from the study subjects before the commencement of the study.

The study included school teachers who provided their consent to participate, as well as those who were employed in the school for at least one academic year and were present during the study period. The teachers who did not give their consent to participate in the study were unavailable during the study period, or who were employed as visiting staff were excluded from the study.

Sample size estimation

A pilot test was conducted among 30 school teachers in Indore city, in which the prevalence of Dental Caries was found to be 76.6%. The relative precision of 3.83% and proportion 76.6% were incorporated into the formula for sample size calculation for cross-sectional study, that is, n = Z^2^P (1-P)/d^2^, where Z is the standard normal variate, P is the expected prevalence and d is the absolute error or precision. The sample size calculated was 462, rounded off to 470 samples.

Simple random sampling was performed using an online random generator tool (https://www.random.org/) to select the schools from the list acquired. The random numbers were generated and corroborated with assigned numbers for schools from the list. Instruments mentioned in WHO Basic Oral Health Survey 5th edition were used, including Mouth Mirror and Community Periodontal Index (CPI) Probe.

Coronavirus disease 2019 (COVID-19) infection control protocol

COVID-19 Infection Control Protocol was followed during the clinical examination and data collection procedure.

Data collection

Data was collected using the WHO Oral Health Assessment Form for Adults. An oral health examination of type III was conducted by an examiner trained and evaluated by VS on a group of 10 patients visiting the Department. The inter-examiner and intra-examiner reliability scores of 0.87 and 0.89 respectively indicated substantial agreement.

Additional information regarding oral hygiene habits, dietary habits, and deleterious habits was assessed using the self-administered modified WHO Oral Health Questionnaire for Adults 2013, which is a pre-tested, pre-validated, structured questionnaire. It included questions on the type of oral hygiene aid used (toothbrush and toothpaste/finger/stick/other) frequency of brushing (once/twice/more than 2 times), the technique of brushing (horizontal/vertical/vibratory/circular), use of fluoridated toothpaste (yes/no/don’t know), tobacco consumption (yes/no) and its form (smokeless/smoke/both) and frequency of intake of specified food items.

The study subjects were imparted individualized oral health education and an appropriate referral system was in place for people requiring dental treatment.

Statistical analysis

The data obtained was entered into Microsoft Excel 2016 and descriptive and inferential statistical analysis was performed using IBM SPSS Statistics for Windows, version 25 (IBM Corp., Armonk, NY) software. Categorical variables were analysed by Chi-square test and continuous variables by One-Way ANOVA. The association between the variables was analysed using an independent t-test and One-Way ANOVA. The confidence interval was fixed at 95%, and α error was set at 5%. A p-value <0.05 was considered statistically significant.

## Results

The current descriptive cross-sectional study reveals the oral health status and treatment needs of school teachers in Indore City. The representative sample was stratified into four age groups of 21-30 years, 31-40 years, 41-50 years, and 51-60 years. The age ranged between 21 and 60 years, with a mean age of 45.32 ± 9.29. Gender distribution revealed 152 (32.3%) teachers were male and 318 (67.7%) teachers were female. The prevalence of smokeless tobacco consumption was 5.1%. All the teachers used toothbrushes and toothpaste. Most of them brushed once a day (72.1%) while 27.9% brushed twice a day. Of the total sample, 35.3% of the representative sample used fluoridated toothpaste, 33.2% did not use fluoridated toothpaste and 31.5% did not know about fluoridated toothpaste (Table 1).

**Table 1:** Demographic details of a representative sample of school teachers in Indore City

Variable	Frequency/n (%)
Gender	
Male	152 (32.3)
Female	318 (67.7)
Age group	
21–30 years	29 (6.2)
31–40 years	118 (25.1)
41–50 years	162 (34.5)
51–60 years	161 (34.3)
Designation	
Primary Teacher (PRT)	102 (21.7)
Trained Graduate Teacher (TGT)	180 (38.3)
Postgraduate Teacher (PGT)	188 (40.0)
Toothbrushing Frequency	
Once a day	336 (71.5)
Twice a day	134 (28.5)
Toothbrushing Technique	
Horizontal	421 (89.6)
Vertical	22 (4.7)
Circular	27 (5.7)
Fluoridated Toothpaste	
Yes	166 (35.3)
No	156 (33.2)
Don’t Know	148 (31.5)
Dietary Habits (items consumed every day)	
Tea with Sugar	433 (92.1)
Coffee with Sugar	134 (28.5)
Sweets/Candies	131 (27.8)

The prevalence of decayed teeth was 68.7% and the caries experience (DMFT) was 82.1%, which showed an increasing trend with an increase in the age group. The prevalence of fluorosis was found to be 2.7% and showed statistically significant differences across the age groups (p<0.001). The prevalence of dental erosion was 3.4%. Around 10.1% showed signs of dental traumatic injuries, of which enamel fracture was the highest (3.2%). The prevalence of oral mucosal lesions was 3.6% and included cases of abscess, oral submucous fibrosis, and koplick spots. Regarding prosthetic status, 1.7% had upper partial dentures and 1.9% had lower partial dentures. The intervention urgency showed that prompt treatment was required by 58.1% of study subjects, followed by preventive or routine treatment (38.9%) (Table 2).

**Table 2:** Prevalence of oral diseases among school teachers of Indore City

Oral Disease	Frequency/n (%)
Decayed Teeth (DT)	323 (68.7)
Cumulative Caries Experience (DMFT)	386 (82.1)
Gingival Bleeding	180 (38.3)
Fluorosis	
Very Mild	11 (2.3)
Mild	1(0.2)
Moderate	1 (0.2)
Erosion	
Enamel Lesion	13 (2.8)
Dentinal	3 (0.6)
Traumatic Injuries	
Treated Injury	10 (2.1)
Enamel Fracture	15 (3.2)
Enamel and Dentine Fracture	6 (1.3)
Pulp Involvement	6 (1.3)
Missing Tooth Due to Trauma	6 (1.3)
Oral Mucosal Lesion	
Abscess	4 (0.8)
Other Conditions	13 (2.8)
Prosthesis	
Upper Removal Partial Denture	8 (1.7)
Lower Removal Partial Denture	9 (1.9)
Intervention Urgency	
Preventive or Routine	183 (38.9)
Prompt	273 (58.1)
Immediate or Urgent	14 (3.0)

Mean Decayed, Missing and Filled Teeth (DMFT) among the representative sample was 3.45 ± 3.10 which showed statistically significant differences across the age groups (p<0.001). The prevalence of shallow pockets was found to be 37.2% and deep pockets were 4.9%. A mean number of 1.20 ± 2.50 and 0.12 ± 0.71 teeth presented with shallow and deep pockets, respectively. The mean number of 4.26 ± 1.97 sextants showed 0-3 mm and 1.50 ± 1.70 sextants showed 4-5 mm attachment loss. A mean number of 0.08 ± 0.37 sextants showed 6-8 mm attachment loss (Table 3).

**Table 3:** Oral health status of a representative sample of school teachers in Indore City *Statistically significant at p<0.05 NS: Not Significant; DMFT: Decayed, Missing and Filled Teeth; LoA: Loss of Attachment

Age Group	Oral Health Status								
	No. of Teeth Present (Mean No. of Teeth ± SD)	DMFT (Mean No. of Teeth ± SD)	Bleeding on Probing (Mean No. of Teeth ± SD)	Healthy Periodontium (Mean No. of Teeth ± SD)	Pocket 4-5 mm (Mean No. of Teeth ± SD)	Pocket ≥6 mm (Mean No. of Teeth ± SD)	LoA 0-3 mm (Mean No. of sextants ± SD )	LoA 4-5 mm (Mean No. of sextants ± SD )	Dental Trauma (Mean No. of Teeth ± SD)
21 – 30 years	30.77 ± 1.42	1.86 ± 1.87	1.52 ± 1.82	30.00 ± 3.11	0.67 ± 2.66	0.00 ± 0.00	5.90 ± 0.31	0.03 ± 0.19	0.21 ± 0.62
31 – 40 years	29.62 ± 1.88	2.31 ± 2.50	1.53 ± 2.50	28.18 ± 3.09	0.93 ± 1.25	0.04 ± 0.24	5.12 ± 1.33	0.75 ± 1.15	0.06 ± 0.30
41 – 50 years	29.94 ± 2.08	3.39 ± 3.25	1.81 ± 2.82	28.16 ± 3.38	1.41 ± 2.23	0.15 ± 0.73	4.35 ± 1.89	1.44 ± 1.60	0.17 ± 0.53
51 – 60 years	29.57 ± 3.27	4.88 ± 3.14	1.23 ± 1.90	27.84 ± 4.72	1.42 ± 2.73	0.17 ± 0.94	3.24 ± 2.09	2.38 ± 1.84	0.13 ± 0.40
Total	30.00 ± 2.44	3.45 ± 3.10	1.52 ± 2.40	28.51 ± 3.91	1.20 ± 2.50	0.12 ± 0.71	4.26 ± 1.97	1.50 ± 1.71	0.13 ± 0.45
P value (ANOVA)	0.000*	0.000*	0.188 (NS)	0.000*	0.043*	0.362 (NS)	0.000*	0.000*	0.185 (NS)

Table 4 shows the association between oral hygiene habits and the mean number of decayed teeth in the study subjects. A significant association was found between the toothbrushing frequency and toothbrushing technique with the mean number of decayed teeth (p<0.05). A higher number of decayed teeth was found among those employing the circular brushing technique. However, no such association was present with the usage of fluoridated toothpaste (p>0.05).

**Table 4:** Association of oral hygiene habits of school teachers with the severity of decayed teeth *statistically significant a: Independent t-test; b: One-Way ANOVA; NS: Not Significant

Toothbrushing Frequency^a^	N	Decayed Teeth (Mean No. of Teeth ± SD)	P value
Once a day	336	2.07 ± 1.85	0.000*
Twice a day	134	0.62 ± 0.72	
Toothbrushing Technique^b^			0.000*
Horizontal	421	1.55 ± 1.75	
Vertical	22	1.55 ± 0.91	
Circular	27	3.33 ± 1.14	
Fluoridated Toothpaste^b^			0.286 (NS)
Yes	166	1.83 ± 1.95	
No	156	1.54 ± 1.47	
Don’t Know	148	1.59 ± 1.75	

## Discussion

Teachers form a bridge between healthcare professionals and the community. An optimal oral health-related knowledge is expected from them, as they are society’s role models. With administratively sound permissions for the smooth running of the present cross-sectional descriptive study on the assessment of oral health status and treatment needs of school teachers in Indore city was undertaken. Modified WHO Oral Health Assessment Proforma was used along with the questionnaire. A higher number of female teachers in comparison were observed. The sole reason could be as teaching is a noble profession and provides convenient work timings too. Teachers from varying backgrounds and levels of educational profiling represented the sample population; diversification in the study due to this was found to be negligible. The gender distribution in the current study is comparable to the study done by Simon et al. [8].

The most popular oral hygiene aid among the representative sample was toothbrush and toothpaste which was similar to that reported by Mota et al.* *[9]. Nevertheless, only 27.9% of our study population brushed twice a day which is lower than reported in their studies. The mean number of decayed teeth was lower in subjects brushing twice a day as compared to those brushing once a day. Our result is in concordance with that reported in a study from Mangalore, India [10].

A dietary assessment revealed that 27.8% of the representative sample consumed sweets and candies daily while tea with sugar was consumed by 92.1%, several times a day. Although they were aware of the maintenance of daily dietary requirements, many of them reported consuming erratic sugar-laden food/drinks frequently.

The prevalence of smokeless tobacco consumption was 5.1% in the current study, whereas there is no report of consumption of the smoking form of tobacco. This finding is lower than the Global School Personnel Survey 2009 for India, where 13.3% of school personnel reported smokeless tobacco consumption [11]. The prevalence is also significantly lower than that reported by Sorensen et al. for school teachers in Maharashtra and Bihar [12].

The prevalence of dental caries in our study was found to be 68.7% probably attributed to the cumulative effect of oral hygiene and dietary habits found amongst the representative sample, as common risk factors responsible for morbidity in all non-communicable diseases. The mean Decayed, Missing and Filled Teeth (DMFT) was 3.45 ± 3.10 in which the filled component contributed significantly indicating escalated oral health care-seeking behavior among school teachers. The prevalence of decayed teeth and mean Decayed, Missing and Filled Teeth (DMFT) in the current study was lower than that reported by Mary et al.in Chennai, India [13]. However, it was higher than that reported among teachers of Davangere [14]. The likely reason for common risk factors associated with oral hygiene practices, diet, non-prioritizing oral health, and lack of knowledge of oral health.

Periodontal status is a direct reflection of oral hygiene practices, which were not found to be practiced fittingly among the study subjects. Gingival bleeding was present among very few study subjects signifying accumulation of chronic condition. The prevalence of shallow periodontal pockets was 37.2% and the mean number of teeth affected was 1.20 ± 2.50, which shows the cumulative effect of the periodontal condition. This was higher than reported by Swamy et al. [15]. Since periodontal condition progresses with age and a greater number of representative samples belonged to the age group of 40-60 years, coupled with improper oral hygiene practices, this could explain the higher prevalence in our study. The prevalence of deep pockets in the current study was 4.9% and the mean number of teeth affected was 0.12 ± 0.71. Female predominance and accompanied hormonal changes in our study sample can be a possible explanation for this finding.

Around 9.1% of school teachers suffered from traumatic injuries to teeth due to accidents and sports injuries. About 2.1% of treated injuries show the vigilance of school teachers towards utilizing dental care treatments, often accessible and affordable by them.

The prevalence of fluorosis in our study was found at 2.7%, with the majority (2.3%) in ‘very mild’ category. Since the teaching profession involves frequent transfers, especially in the government sector, the prevalence of fluorosis could be due to migratory reasons. The presence of fluoride belts in Madhya Pradesh could also explain the present prevalence of fluorosis [16].

The current study showed a prevalence of 3.6% of oral mucosal lesions. The findings were higher than that reported in Chennai [13]. The findings of the oral mucosal lesion could be stress-induced or be attributed to the prevalence of the smokeless tobacco habit. There could also be nutritional deficiencies among the study subjects as oral mucosal lesions result from abnormal intake of vitamins and minerals, as reported by Thomas et al. [17].

The prosthetic status in a population signifies the oral health treatment-seeking habit and the awareness to utilize the provision of dental services. In the present study, 1.7% and 1.9% of the teachers had upper and lower removable partial dentures respectively. This could be because our population comprised of society’s educated segment and hence oral health care seeking was high among them. Our findings are in concordance with other similar studies [13-14].

The need for preventive or routine treatment was 38.9% while 58.1% required prompt treatment, which reveals a lack of mindset due to unawareness regarding appropriate knowledge of oral health. Immediate or urgent treatment was indicated for 3% of the examined study sample due to silent non-symptomatic morbid conditions. Since the majority of school teachers overlook on importance of oral health, the preventive treatment for optimum oral health stands trivial. Only symptomatic treatment was pursued and given importance by them as subjected to disruption of routine. Consequently, the need for prompt and preventive treatment for oral health promotion among the representative sample needs to be addressed on prime precedence. The findings are in concordance with the intervention urgencies reported by other studies [15].

The study recommends the implementation of the Teachers Training Program as formulated by the Dental Council of India in their Publication “Training Manual on Oral Health Promotion for School Teachers” [18]. The role of school teachers is vital for the initiative targeting school children since school is often the only environment equipped with the resources to expose children to healthy practices. Integrated program approach where oral health-related knowledge including common oral diseases, the importance of prevention in oral health, and common myths and facts regarding oral health needs to be imparted. School Teachers can also act as an important link between educational institutes and oral healthcare providers.

## Conclusions

The present study revealed that the study subjects showed an overall oral health status with mixed results. While the caries experience was high among the sample, it also included a high proportion of filled teeth, which is a positive sign of their oral healthcare-seeking habits. The prevalence of gingival and periodontal disease necessitates the provision of promotive, preventive, and curative oral health care services for school teachers. In an effort to target the oral health of the school community by taking a holistic course of action, it is imperative that oral hygiene knowledge and associated practices be alleviated among the school teachers. Our results were found to be in concordance with other studies conducted in various parts of India on school teachers.
